# Efficacy and Safety of Novel Oral Anti-Cholestatic Agents for Primary Biliary Cholangitis: Meta-Analyses and Systematic Review

**DOI:** 10.3390/ph18050697

**Published:** 2025-05-08

**Authors:** Eyad Gadour, Bogdan Miutescu, Hiba Bashir, Abubaker Ali, Salem Alanzi, Abdullah A. Al-Shahrani, Aymen Almuhaidb, Shahed Mohamed, Faisal Abaalkhail, Hadi Kuriry, Mohammed Saad AlQahtani

**Affiliations:** 1 Multiorgan Transplant Centre of Excellence, Liver Transplant Unit, King Fahad Specialist Hospital, Dammam 32253, Saudi Arabia; h.kuriry@live.com (H.K.); alqahtanim222@gmail.com (M.S.A.); 2 Department of Medicine, Zamzam University College, Khartoum 11113, Sudan; 3 Department of Gastroenterology and Hepatology, “Victor Babes” University of Medicine and Pharmacy, 300041 Timisoara, Romania; bmiutescu@yahoo.com; 4 Advanced Regional Research Centre in Gastroenterology and Hepatology, “Victor Babes” University of Medicine and Pharmacy, 300041 Timisoara, Romania; 5 Gastroenterology, Yas Clinic Abu Dhabi Stem Cell Center, Abu Dhabi SE44, United Arab Emirates; drhibabeshir@yahoo.com; 6 Department of Medicine, King Abdulaziz National Guard Hospital, Ahsa 36428, Saudi Arabia; abubaker6699@hotmail.com (A.A.); salemalanizi90@gmail.com (S.A.); 7 Gastroenterology, King Khalid University, Abha 62521, Saudi Arabia; dr.ashahrani@outlook.com; 8 Department of Gastroenterology, King Faisal Specialist Hospital and Research Center, Riyadh 11211, Saudi Arabia; aalmuhaidb@kfshrc.edu.sa; 9 Department of Medicine, Icahn School of Medicine at Mount Sinai, New York, NY 10029, USA; drshahedmohamed@gmail.com; 10 College of Medicine, Alfaisal University, Riyadh 11211, Saudi Arabia; faisal.abaalkhail@gmail.com; 11 Department of Liver and Small Bowel Transplantation and Hepatobiliary-Pancreatic Surgery, King Faisal Specialist Hospital and Resesarch Centre, Riyadh 11211, Saudi Arabia; 12 Department of Surgery, Imam Abdulrahman Bin Faisal University, Dammam 31441, Saudi Arabia

**Keywords:** primary biliary cholangitis, ursodeoxycholic acid, anti-cholestatic agents, novel therapies, alkaline phosphatase, pruritus, systematic review, meta-analysis

## Abstract

**Background:** Primary biliary cholangitis (PBC) is a chronic autoimmune liver disease characterized by progressive bile duct damage and cholestasis. While ursodeoxycholic acid (UDCA) is the first-line therapy, approximately 40% of patients have incomplete responses, necessitating alternative treatments. This systematic review and meta-analysis evaluate the efficacy and safety of novel oral anti-cholestatic agents for PBC. **Methods:** A systematic literature search was conducted in electronic databases up to September 2024. Randomized controlled trials, cohort studies, and case-control studies evaluating novel oral anti-cholestatic agents in adult PBC patients were included. The primary outcome was a change in alkaline phosphatase (ALP) levels. Safety was assessed by the incidence of serious adverse events. Random-effect meta-analyses were performed. **Results:** Ten studies involving 878 patients were analyzed. Novel agents included seladelpar, fenofibrate, saroglitazar, bezafibrate, elafibranor, and budesonide. The meta-analysis showed significant reductions in ALP levels with novel agents compared to the controls (SMD −2.80; 95% CI −3.56, −2.03; *p* < 0.00001), with high heterogeneity (I^2^ = 93%). Saroglitazar achieved the largest effect size. There was no significant difference in serious adverse events between novel agents and controls (OR 1.21; 95% CI 0.81, 1.83; *p* = 0.35). **Conclusions:** Novel oral anti-cholestatic agents show promise in improving biochemical markers in PBC patients with suboptimal UDCA responses, with a safety profile comparable to controls. However, study heterogeneity and limited long-term data restrict direct comparisons. Larger standardized trials with extended follow-up are needed to confirm long-term efficacy and safety.

## 1. Introduction

Primary biliary cholangitis (PBC) is a chronic autoimmune liver disease that causes damage to the intrahepatic bile ducts, leading to cholestasis and potentially progressing to cirrhosis and liver failure [1,2]. PBC is rare, with an incidence ranging from 0.33 to 5.8 per 10,000 people annually and a prevalence between 1.91 and 40.2 per 100,000 individuals [1,3,4,5]. The disease primarily affects middle-aged women, aged 40–70 years, with symptoms ranging from mild elevations in cholestatic liver enzymes to severe fatigue, pruritus, and complications of liver damage. Epidemiological studies show a strong female predominance with a sex ratio of 9:1 [3,4]. However, recent data indicate an increasing incidence in adult men, reducing the female-to-male ratio to 4–6:1 [6,7,8].

The exact cause of PBC is still unclear, but research suggests it involves a combination of genetic factors and environmental triggers [1]. Studies have highlighted the role of epigenetics in disease development, with Selmi et al. [9] reporting a 63% concordance in identical twins and a 9.13–10.5 relative risk among first-degree relatives [10]. Molecular mimicry between lipoic acids, xenobiotics, and exogenous antigens is also thought to contribute to the loss of tolerance in biliary epithelial cells, triggering immune responses associated with the disease [11,12,13].

Clinically, PBC often presents without symptoms initially, with fatigue (experienced by up to 80% of patients) and pruritus on the palms and soles being common early signs [14]. These symptoms are strongly linked to poor quality of life [15,16]. The treatment approach for PBC has advanced over time. Ursodeoxycholic acid (UDCA) remains the first-line therapy, offering significant improvements in liver function, slowing disease progression, and increasing survival, without the need for liver transplantation. According to Lammers et al. (2014), UDCA treatment has been associated with survival rates of 90%, 78%, and 66% at 5, 10, and 15 years, respectively [17]. However, around 40% of patients experience inadequate biochemical responses to UDCA, requiring alternative or additional treatments [13]. Nova oral anti-cholestatic agents are becoming significant in the management of cholestatic liver diseases, particularly primary biliary cholangitis (PBC). These novel therapies present diverse mechanisms of action distinct from the traditional treatment with ursodeoxycholic acid (UDCA). The mechanisms of action for each of the specified agents, including seladelpar, fenofibrate, saroglitazar, bezafibrate, elafibranor, and budesonide, are explained in detail as follows. Seladelpar is a selective agonist of the peroxisome proliferator-activated receptor delta (PPAR-δ), which plays a pivotal role in lipid metabolism, inflammation, and cellular differentiation. By activating PPAR-δ, seladelpar enhances fatty acid oxidation and mitigates liver inflammation and fibrosis. This mechanism is particularly advantageous in primary biliary cholangitis (PBC), where cholestasis results in liver injury and inflammation. The selective action of seladelpar on PPAR-δ enables it to effectively modulate lipid metabolism and inflammatory pathways, potentially leading to improved liver function and histological outcomes in patients with PBC.

Fenofibrate functions primarily as an agonist of the PPAR- α receptor, which plays a crucial role in the regulation of lipid metabolism, particularly in the catabolism of fatty acids. Fenofibrate facilitates the oxidation of fatty acids and reduces triglyceride levels, thereby potentially alleviating cholestatic conditions. In the context of primary biliary cholangitis (PBC), fenofibrate’s capacity to enhance lipid metabolism may mitigate liver inflammation and improve bile flow, thus addressing some of the underlying mechanisms of cholestasis.

Saroglitazar functions as a dual agonist for PPAR- α and PPAR- γ. The activation of PPAR- α enhances lipid metabolism and reduces triglyceride levels, akin to the effects of fenofibrate. Concurrently, PPAR- γ activation is linked to improved insulin sensitivity and exhibits anti-inflammatory properties. This dual mechanism enables saroglitazar to address both the metabolic and inflammatory aspects of primary biliary cholangitis (PBC), potentially facilitating the enhanced management of cholestatic liver disease by improving liver function and mitigating inflammation.

Bezafibrate is a fibrate that functions as an agonist for multiple peroxisome proliferator-activated receptor (PPAR) subtypes, including PPAR- α, PPAR- β, and PPAR- γ. Its broad-spectrum activity enables it to modulate various metabolic pathways, such as lipid metabolism, glucose homeostasis, and inflammation. In the context of primary biliary cholangitis (PBC), bezafibrate may contribute to reducing bile acid toxicity, improving lipid profiles, and exerting anti-inflammatory effects, thereby serving as a versatile therapeutic option for managing cholestasis.

Elafibranor is a dual agonist that targets both PPAR- α and PPAR- δ. This dual mechanism facilitates enhanced lipid metabolism and exerts anti-inflammatory effects. Elafibranor has the potential to ameliorate features of metabolic syndrome commonly associated with cholestatic liver diseases, thereby addressing multiple aspects of the pathophysiology of primary biliary cholangitis (PBC). By promoting liver health and reducing inflammation, elafibranor may improve overall outcomes for patients with PBC.

Budesonide, a corticosteroid, has been repurposed for the treatment of primary biliary cholangitis (PBC) due to its distinctive mechanism of action. It effectively downregulates pro-inflammatory cytokines, including interleukin-1 (IL-1), interleukin-6 (IL-6), and tumor necrosis factor-alpha (TNF-alpha), as well as chemokines involved in the inflammatory response. By addressing the inflammatory and immune dysregulation associated with PBC, budesonide can provide symptomatic relief and enhance liver function. Its favorable safety profile and potential for combination therapy render it a promising therapeutic option [18,19,20].

This systematic review and meta-analysis aimed to compare the efficacy and safety of novel oral agents in patients with PBC. This study aimed to support clinical decision-making, guide future research, and improve patient outcomes by providing a thorough evaluation of their therapeutic effects.

## 2. Methodology

### 2.1. Study Design

This systematic review adhered strictly to the Preferred Reporting Items for Systematic Reviews and Meta-Analyses (PRISMA) guidelines, ensuring methodological rigor and transparency throughout the process. The PRISMA statement, a widely accepted set of standards for reporting systematic reviews and meta-analyses, was meticulously followed to enhance the quality and clarity of the review. As this study exclusively utilized published data and did not involve direct human participant interaction, ethical approval was not required.

A thorough literature search was performed across several electronic databases, including PubMed, Embase, Cochrane Library, and Web of Science, covering studies from their inception to September 2024. The search used MeSH terms and keywords related to “primary biliary cholangitis”, “anti-cholestatic agents”, “oral therapies”, and specific agents like “obeticholic acid” and “fibrates”. Additionally, grey literature, conference proceedings, and clinical trial registries were reviewed to identify unpublished studies. Study characteristics and outcomes were tabulated using Microsoft Excel. Forest plots and funnel plots for meta-analyses were generated using Review Manager (RevMan 7.2.0) software. This article follows the PICOS framework, addressing population (adult patients with primary biliary cholangitis), interventions (novel oral anti-cholestatic agents), comparators (placebo, UDCA monotherapy, or other oral anti-cholestatic agents), outcomes (efficacy and safety measures), and study designs (randomized controlled trials, cohort studies, and case-control studies) in the selection and analysis of included studies (Table 1). Random-effect meta-analyses were performed. The review was registered in PROSPERO (CRD420251031204).

### 2.2. Eligibility Criteria

This meta-analysis used the population, intervention, comparison, and outcomes framework to select the most relevant studies for inclusion. Studies were eligible if they met the following criteria.

- Population: Adult patients diagnosed with PBC using established diagnostic criteria;
- Intervention: Novel oral anti-cholestatic agents (e.g., obeticholic acid, fibrates, nor-UDCA, etc.);
- Comparator: Placebo, UDCA monotherapy, or other oral anti-cholestatic agents;
- Outcomes: Efficacy (e.g., improvements in alkaline phosphatase [ALP], bilirubin levels, or pruritus) and safety (e.g., adverse events, tolerability);
- Study Design: Randomized controlled trials (RCTs), cohort studies, or case-control studies.

Studies were excluded if they were non-human or case reports and if they lacked quantitative data.

### 2.3. Data Extraction and Quality Assessment

Two independent reviewers screened titles, abstracts, and full texts to determine their eligibility. Any disagreements were resolved by discussion or consultation with a third reviewer. A standardized data extraction form was used to gather information on the study characteristics, population demographics, interventions, comparators, outcomes, and follow-up periods.

The risk of bias in each study was assessed using the Cochrane risk-of-bias tool. Bias was assessed in areas such as sequence generation, allocation concealment, participant and personnel blinding, outcome assessment blinding, incomplete outcome data, selective reporting, and other potential biases.

### 2.4. Data Synthesis and Statistical Analysis

Data analysis was conducted using the Review Manager (RevMan) for advanced statistical techniques. A random effect model was used in the meta-analysis to account for potential heterogeneity. Pooled effect sizes for continuous outcomes (e.g., ALP reduction) were reported as mean differences with 95% confidence intervals (CIs). Odds ratios (ORs) with 95% CIs were calculated for dichotomous outcomes (e.g., adverse event incidence). Heterogeneity was assessed using the I^2^ statistic with low (I^2^ < 25%), moderate (I^2^ = 25–50%), and high (I^2^ > 50%) thresholds. Publication bias was examined using funnel plots and Egger’s test.

## 3. Results

### 3.1. Literature Search

**Figure 1:**
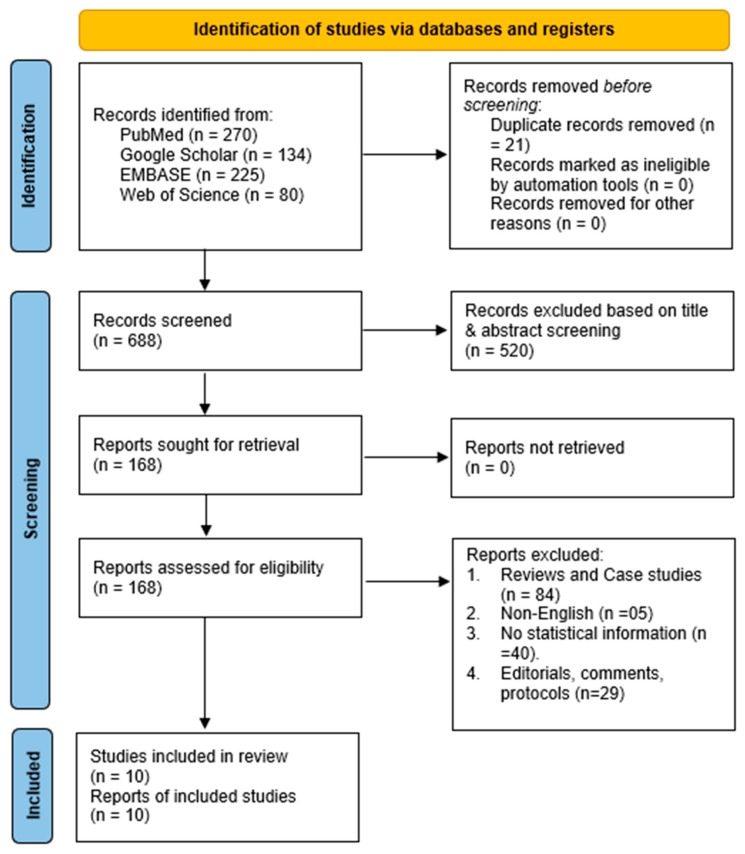
PRISMA flow diagram illustrating the process of literature identification and selection.

The literature search yielded 709 records from electronic databases. After removing 21 duplicates, 688 articles were screened based on their titles and abstracts, resulting in the exclusion of 520 irrelevant records. This process resulted in 168 studies being retrieved for further evaluation, all of which were assessed against predefined eligibility criteria. Of these, 158 studies were excluded for the following reasons: 84 were reviews or case studies, five were languages other than English, 40 lacked essential statistical data, and 29 were editorial, protocol, or comment articles. Finally, ten studies met the eligibility criteria and were included in the review. Figure 1 shows the study identification process using the databases searched.

### 3.2. Study Characteristics

This analysis included 10 studies with a total of 878 patients, evaluating the efficacy and safety of various anti-cholestatic agents for treating PBC [21,22,23,24,25,26,27,28,29,30]. The studies comprised eight placebo-controlled and open-label studies and two retrospective studies from regions including China, the USA, Europe, and multinational cohorts (Table 2). The sample sizes ranged from 37 to 265 patients, and the study duration varied from 2 weeks to 24 months. Several interventions, such as saroglitazar and fenofibrate, showed significant rates of ALP normalization, with some exceeding 50%. Improvements in pruritus, a common and debilitating symptom of PBC, have also been observed in several trials, highlighting the potential of these agents to improve both biochemical and symptomatic outcomes for PBC patients with PBC. Table 3 shows a summary table of quality ratings for all studies.

### 3.3. Efficacy of Novel Cholestatic Drugs

**Figure 2:**
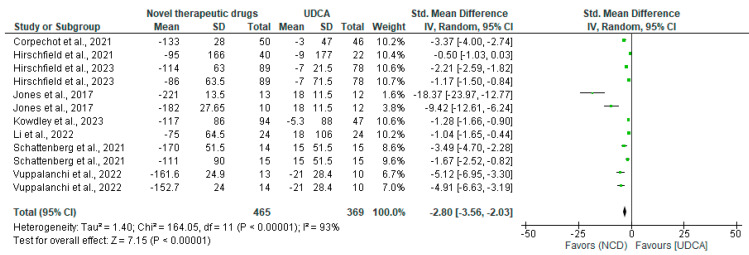
Changes in ALP levels in PBC patients treated with NCDs compared to controls.

The analysis of novel cholestatic drugs (NCDs) demonstrated a significant impact on alkaline phosphatase (ALP) levels, a key biomarker for cholestatic liver diseases. The standardized mean difference (SMD) of −2.80, with a 95% confidence interval of [−3.56, −2.03] and a *p*-value < 0.00001, indicates a substantial and statistically significant reduction in ALP levels compared to control drugs (Figure 2). This reduction in ALP is clinically important as elevated ALP levels are characteristic of cholestatic conditions. The decrease suggests that NCDs may effectively improve bile flow and reduce liver damage associated with cholestasis. The magnitude of the effect (SMD −2.80) implies a large clinical benefit, potentially translating to improved liver function and patient outcomes.

However, the high heterogeneity (93%) observed in the results warrants caution in interpretation. This heterogeneity suggests variability in the effect sizes across different studies, which could be due to factors such as differences in study designs, patient populations, or specific drug mechanisms (Table 4). The sensitivity analysis, which attempted to account for differences in drugs used across studies, did not resolve the heterogeneity issue. This persistent heterogeneity implies that other factors beyond drug type may be influencing the results, such as disease severity, duration of treatment, or patient characteristics. Interestingly, saroglitazar stood out as the only treatment without heterogeneity, likely due to data coming from a single three-arm study with consistent conditions. This consistency in saroglitazar’s results provides more reliable evidence for its effect on ALP levels, though it may limit generalizability to broader patient populations. The focus on ALP as the primary efficacy measure across most studies underscores its importance in evaluating cholestatic drug efficacy. However, the variation in outcome reporting between studies highlights the need for standardized reporting in future research to facilitate more comprehensive meta-analyses and clearer clinical guidelines for cholestatic disease management.

**Figure 3:**
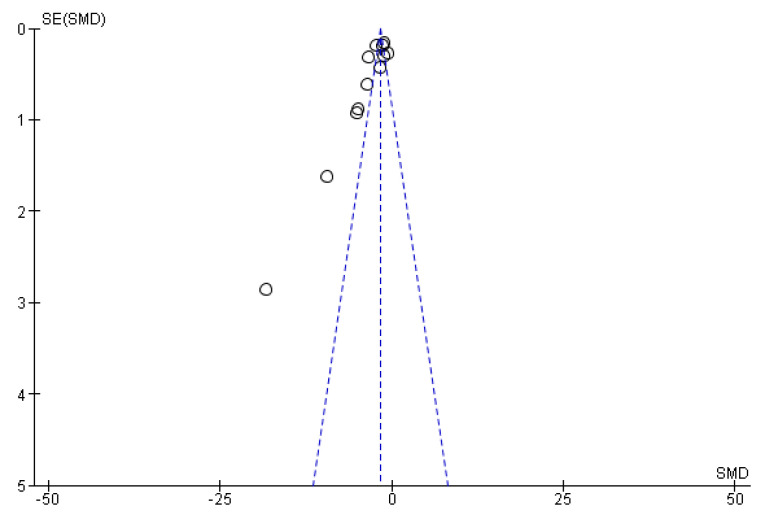
Funnel plot for publication bias in studies assessing changes in ALP levels in PBC patients treated with NCDs versus controls.

The funnel plot below assesses publication bias by evaluating changes in ALP levels in patients with PBC treated with NCDs versus controls (Figure 3). The plot shows some asymmetry, especially at the lower end, where smaller studies with higher standard errors deviate from the overall trend. This suggests possible publication bias or heterogeneity, as smaller studies with non-significant results may be underrepresented, or there could be methodological differences. While the pooled results are supported by larger studies, the observed asymmetry indicates the need for caution and further investigation of potential biases and heterogeneity.

### 3.4. Safety

**Figure 4:**
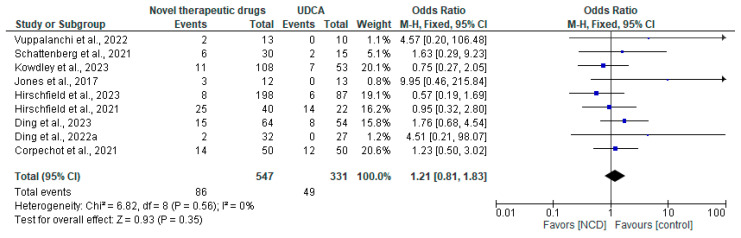
Forest plot showing the safety outcomes of NCDs in PBC patients compared to controls.

Nine studies reported the safety outcomes of NCD use in patients with PBC. Our analysis showed no significant difference in the incidence of serious adverse events between patients treated with NCDs and those in the control group (OR, 1.21; 95% CI [0.81, 1.83]; *p* = 0.35). There was no heterogeneity across the studies I^2^ = 0% (Figure 4).

**Figure 5:**
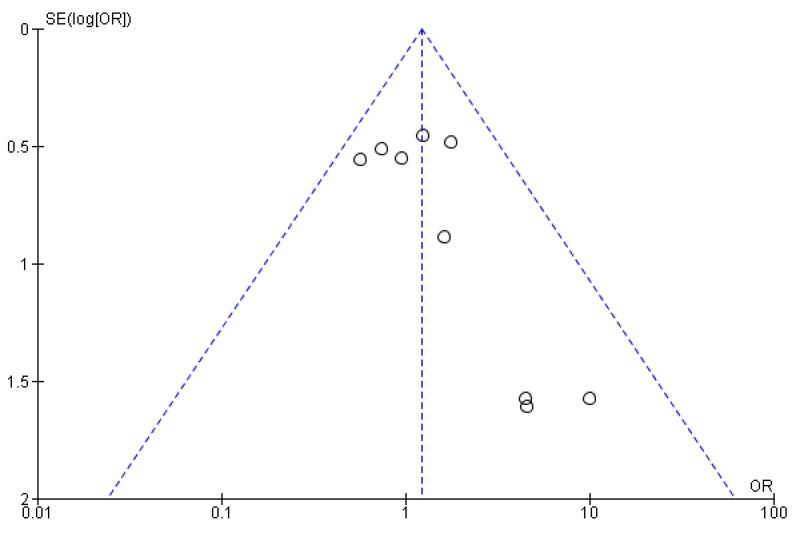
Funnel plot assessing safety outcomes in PBC patients treated with NCDs versus controls.

The funnel plot for safety outcomes in patients with PBC treated with NCDs versus controls (Figure 5) showed a symmetrical distribution around OR = 1, suggesting minimal publication bias. Larger studies are clustered near the top, with a few outliers indicating potential heterogeneity.

### 3.5. Risk of Bias Assessment

**Figure 6:**
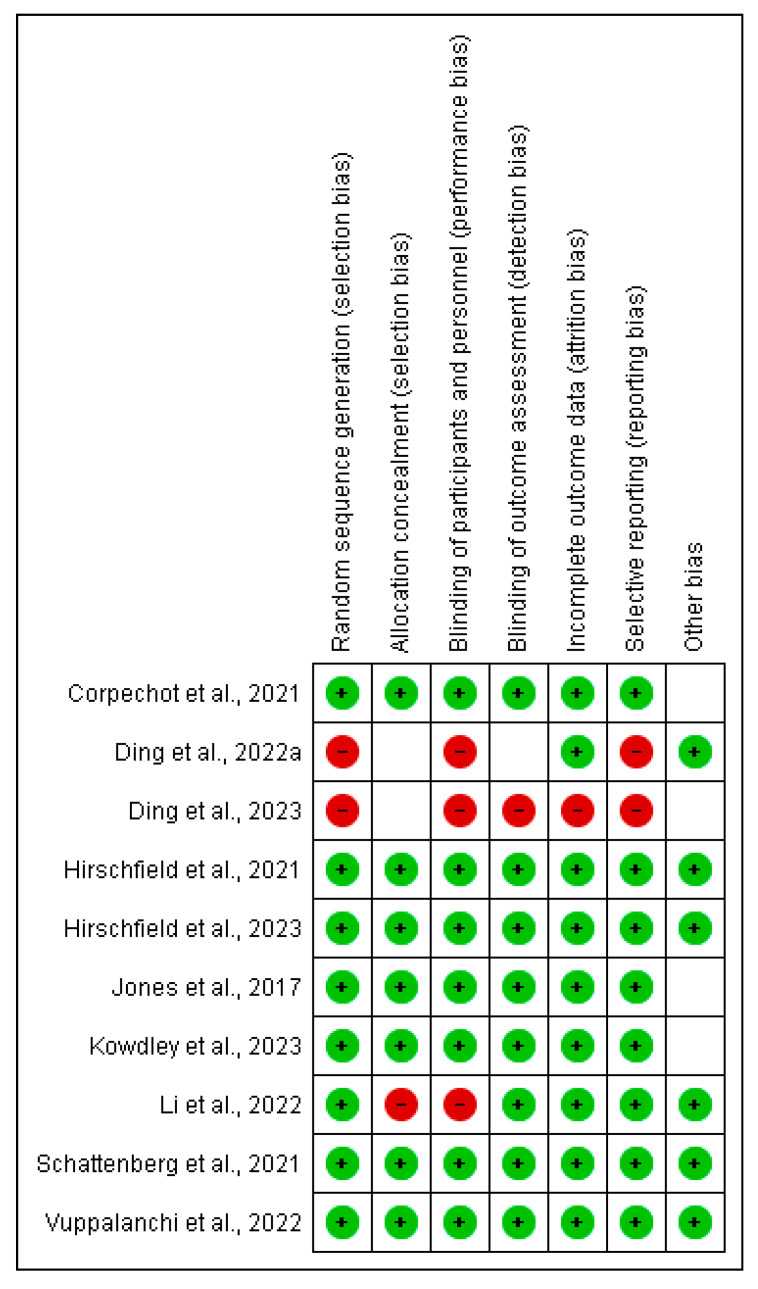
Risk of bias plot for each study.

Overall, most studies showed a low risk of bias in most areas, as indicated by the predominance of the green circles (Figure 6). The handling of incomplete outcome data and selective reporting is generally well managed, suggesting that missing data and selective reporting did not significantly affect the results. Other potential biases, such as those related to funding or conflicts of interest, appear to be minimal, with most studies showing low risk in these areas.

## 4. Discussion

This systematic review and meta-analysis evaluated the efficacy and safety of NCDs for PBC, emphasizing their potential as treatment options for patients who do not respond adequately to UDCA. Our results indicate that NCDs significantly lowered ALP levels, a key indicator of disease progression, although the effect sizes varied among the different drugs. Safety outcomes, measured by the incidence of serious adverse events, were similar between NCDs and control treatments, supporting their potential as alternative or supplementary therapies in PBC management.

In gastroenterology, the reduction of ALP levels is widely recognized as a crucial indicator of improved outcomes in PBC. This reduction is associated with lower risks of hepatic decompensation and a decreased likelihood of requiring liver transplantation, as evidenced by numerous studies in the field [17,18].

The significance of ALP reduction extends beyond PBC, as it serves as a key marker for assessing the effectiveness of treatments in various cholestatic liver diseases. Rupp’s analysis of novel cholestatic drugs (NCDs) revealed a significant reduction in ALP levels across all included treatments, underscoring their efficacy in alleviating cholestasis [31]. This finding is particularly important as it suggests that these NCDs are effectively targeting the underlying mechanisms of cholestatic liver diseases, potentially offering new therapeutic options for patients who may not respond well to conventional treatments. Among the NCDs studied, saroglitazar, a peroxisome proliferator-activated receptor (PPAR) antagonist, demonstrated the most substantial effect size (SMD = −5.01) with no heterogeneity [32]. This remarkable consistency in results positions saroglitazar as a highly promising treatment option for cholestatic liver diseases. The uniformity of its effects may be attributed to the controlled conditions of the single study from which these results were derived, including standardized dosing protocols and carefully selected patient demographics. However, it is crucial to note that the promising efficacy of saroglitazar is balanced by reports of adverse events leading to treatment discontinuation. This observation aligns with findings reported by Bernal et al. [32].

In contrast, drugs such as seladelpar (a selective PPAR- δ agonist) and elafibranor showed greater variability (I^2^ = 96% and 83%, respectively), which may reflect differences in patient populations, baseline disease severities, or study designs. Moreover, the early termination of the Jones et al. [22] trial and safety concerns noted in the ENHANCE [25] study create some uncertainty about the safety and efficacy of seladelpar [33]. Despite these issues, the overall trend supports their potential utility, though further analyses are needed to identify the patient subgroups most likely to benefit, with larger long-term studies being necessary [27]. Additionally, certain drugs, such as budesonide, did not show a statistically significant reduction in ALP, raising doubts about their effectiveness in treating PBC [34].

Based on nine studies, the safety profile of NCDs was similar to that of controls, with no significant increase in serious adverse events (OR, 1.21; *p* = 0.35) and no heterogeneity between studies (I^2^ = 0%). This is reassuring, especially considering concerns regarding the long-term use of new medications. However, the short follow-up periods in these studies may have limited the detection of rare or delayed adverse effects such as hepatotoxicity or systemic side effects. For example, while fibrates are effective at reducing ALP, they carry potential risks of renal impairment, requiring careful monitoring in clinical settings.

## 5. Limitations

Although the studies included in this analysis provide valuable insights into anti-cholestatic therapies for PBC, they have several limitations. First, some trials had small sample sizes such as 37 patients and 45 patients which may limit the generalizability of the results [23,27]. Second, the study durations varied considerably, with some lasting only 12 weeks, potentially missing long-term efficacy and safety data [22,27]. Conversely, longer trials, such as those conducted for 24 months and 32.9 months offer valuable information, but their observational nature could introduce bias compared with placebo-controlled designs [13,25]. Additionally, there was variability in the study endpoints, with some focusing on ALP normalization and others on pruritus improvement [21,22]. The inconsistent reporting of pruritus limits conclusions regarding symptom relief [21,22,23]. Furthermore, not all studies have thoroughly reported adverse events, leaving gaps in safety evaluations. Finally, the geographic diversity of the patient populations (e.g., China, Europe, and the USA) may affect the external validity of the findings due to differences in genetic, environmental, or healthcare systems.

## 6. Future Directions

Future research should prioritize larger multicenter RCTs with standardized endpoints, assessing both biochemical markers and symptomatic improvements. Extended follow-up periods are crucial to evaluate long-term efficacy and safety. Head-to-head comparisons of novel agents like seladelpar, fenofibrate, and saroglitazar are necessary to identify optimal treatments for PBC. Investigating these agents’ mechanisms of action could reveal biomarkers for predicting treatment response, enabling personalized therapies. Including diverse patient populations would enhance the result generalizability. Cost-effectiveness analyses are essential for clinical practice considerations. Exploring combination therapies, such as UDCA with seladelpar or bezafibrate, may uncover synergistic effects benefiting non-responders. These approaches will address current limitations and advance PBC treatment strategies.

## 7. Conclusions

This review highlights the potential of novel anti-cholestatic agents in the treatment of PBC. The therapies examined, including fenofibrate, seladelpar, saroglitazar, bezafibrate, elafibranor, and budesonide, showed significant reductions in ALP levels, with notable ALP normalization and symptomatic relief from pruritus in several trials. However, the variation in study designs, sample sizes, and endpoints calls for further large-scale research to determine the optimal use of these agents. While these therapies show promise for improving patient outcomes, consistent reporting of both biochemical and symptomatic endpoints, as well as safety data, is essential. As research continues, integrating these therapies into PBC treatment guidelines will depend on their proven efficacy, safety, and cost-effectiveness. Ultimately, a deeper understanding of PBC pathophysiology and therapeutic responses will support the development of targeted, patient-focused treatment strategies to improve quality of life and clinical outcomes for this complex disease.

## Figures and Tables

**Table 1:** Outlining the PICOS framework for each study.

Study	Population	Intervention	Comparator	Outcomes	Study Design
Ding et al., 2023 [21]	Adult patients with PBC	Fenofibrate	UDCA	ALP levels, long-term outcomes	Retrospective
Jones et al., 2017 [22]	Adult patients with PBC	Seladelpar (50 mg, 200 mg)	Placebo	ALP reduction, normalization rates	Phase 2 RCT
Vuppalanchi et al., 2022 [23]	Adult patients with PBC	Saroglitazar (2 mg, 4 mg)	Placebo	ALP levels, normalization rates	Phase 2 RCT
Li et al., 2022 [24]	Adult patients with PBC	Fenofibrate	Placebo	ALP levels	Open-label RCT
Hirschfield et al., 2023 [25]	Adult patients with PBC	Seladelpar (5 mg, 10 mg)	Placebo	ALP levels, safety outcomes	Phase 3 RCT
Kowdley et al., 2024 [26]	Adult patients with PBC	Elafibranor (80 mg)	Placebo	ALP levels, safety outcomes	Phase 3 RCT
Schattenberg et al., 2021 [27]	Adult patients with PBC	Elafibranor (80 mg, 120 mg)	Placebo	ALP levels, safety outcomes	Phase 3 RCT
Ding et al., 2022 [28]	Adult patients with advanced PBC	Fenofibrate	UDCA	ALP levels	Retrospective
Corpechot et al., 2018 [29]	Adult patients with PBC	Bezafibrate (400 mg)	Placebo	ALP levels, normalization rates	Phase 3 RCT
Hirschfield et al., 2021 [30]	Adult patients with PBC	Budesonide	Placebo	ALP levels, pruritus improvement	Phase 3 RCT

**Table 2:** Characteristics of the included studies.

Author ID	Design	Country	NCD	N	Randomization			Study Duration	Outcomes								
									ALP Changes (U/l)			ALP Normalization			Pruritus		
Ding et al., 2023 [21]	Retrospective	China	Fenofibrate	118	Fenofibrate	UDCA		36 (24–48) weeks	Fenofibrate		UDCA	Fenofibrate	UDCA		Not reported		
					64	54			29		14	24	6				
Jones et al., 2017 [22]	Phase 2 RCT	North America and Europe	Seladelpar	41	Seladelpar (50 g)	Seladelpar (200 mg)	Placebo	12 weeks	Seladelpar (50 g)	Seladelpar (200 g)	Placebo	Seladelpar (50 g)	Seladelpar (200 g)	Placebo	Seladelpar (50 g)	Seladelpar (200 g)	Placebo
					13	13	12		53% (14)	63% (8)	−2% (16)	100%	100%	0	4	1	1
									**Reduction in ALP levels by at least 15%**			**ALP normalization**					
Vuppalanchi et al., 2022 [23]	Phase 2 RCT	USA	Saroglitazar	37	Saroglitazar (4 mg)	Saroglitazar (2 mg)	Placebo	16 weeks	Saroglitazar (4 mg)	Saroglitazar (2 mg)	placebo	Saroglitazar (4 mg)	Saroglitazar (2 mg)	Placebo	Not reported		
					13	14	10		84.60%	92.90%	20%	38.50%	50%	0%			
Li et al., 2022 [24]	Open-label RCT	China	Fenofibrate	48	Fenofibrate		Placebo	58 weeks	Not reported			Fenofibrate		Control	Not reported		
					24		24					54.20%		4.2%			
Hirschfield et al., 2023 [25]	Phase 3 RCT	Multinational (21 countries)	Seladelpar	265	Seladelpar (5 mg)	Seladelpar (10 mg)	Placebo	58 weeks	Not reported			Seladelpar (5 mg)	Seladelpar (10 mg)	placebo	Seladelpar (5 mg)	Seladelpar (10 mg)	Placebo
					89	89	87					5.40%	27.30%	0%	3.40%	11.20%	12.60%
Kowdley et al., 2024 [26]	Phase 3 RCT	Multinational	Elafibranor	161	Elafibranor (80 mg)		Placebo	52 weeks	Elafibranor (80 mg) LS		Placebo	Elafibranor (80 mg)		Placebo	Elafibranor (80 mg)		Placebo
					108		53		−117.0 (−134.4–−99.6)		−5.3 (−30.4–19.7)	15%		0%	20%		26%
Shattenberg et al., 2021 [27]	Phase 3 RCT	USA and Europe	Elafibranor	45	Elafibranor (80 mg)	Elafibranor (120 mg)	Placebo	12 weeks	Elafibranor (80 mg)	Elafibranor (120 mg)	Placebo	Elafibranor (80 mg)	Elafibranor (120 mg)	Placebo	Elafibranor (80 mg)	Elafibranor (120 mg)	Placebo
					15	15	15		−48.3 ± 14.8%	−40.6 ± 17.4%	+3.2 ± 14.8%	13.30%	21.40%	0%	0	0	2
Ding et al., 2022 [28]	Retrospective	China	Fenofibrate	59	Fenofibrate	N/A	UDCA monotherapy	24 months	Fenofibrate		Placebo	Fenofibrate	UDCA		Not reported		
					32	N/A	27		25		23	10	0				
									**Median change from baseline**								
Corpechot et al., 2018 [29]	Phase 3 RCT	France	Bezafibrate	100	Bezafibrate (400 mg)		Placebo	24 months	Bezafibrate		Placebo	Bezafibrate		Placebo	Bezafibrate		Placebo
					50		50		−60 (−66–−46)		0 (−14–20)	67%		2%	8%		14%
									**Mean change from baseline** **(SD)**						**Change from baseline**		
Hirschfield et al., 2021 [30]	Phase 3 RCT	Multinational	Budesonide	62	Budesonide		Placebo	32.9 months	Budesonide		Placebo	Budesonide		Placebo	Budesonide		Placebo
					40		22		ç95 (166)		−9 (177)	35%		9%	−0.3 (2.3)		0.6 (3.1)

N (n), mean (SD); N (n1-n2), median (IQR); LS, least squares; UDCA, ursodeoxycholic acid; RCT, randomized controlled trial.

**Table 3:** Quality ratings summary for all studies.

Study	Study Design	Quality Rating	Comments
Ding et al., 2023 [21]	Retrospective	Low	Limited sample size, potential bias in data collection.
Jones et al., 2017 [22]	Phase 2 RCT	Low	Early termination of the trial, safety concerns noted.
Vuppalanchi et al., 2022 [23]	Phase 2 RCT	Moderate	Small sample size, but clear outcome measures.
Li et al., 2022 [24]	Open-label RCT	Moderate	Short duration, but significant findings on ALP.
Hirschfield et al., 2023 [25]	Phase 3 RCT	Low	Potential bias due to early termination and safety concerns.
Kowdley et al., 2024 [26]	Phase 3 RCT	Moderate	Larger sample size, but variability in patient demographics.
Schattenberg et al., 2021 [27]	Phase 3 RCT	Moderate	Short duration, but clear outcome measures.
Ding et al., 2022 [28]	Retrospective	Low	Limited sample size, potential bias in data collection.
Corpechot et al., 2018 [29]	Phase 3 RCT	Moderate	Larger sample size, but variability in outcome measures.
Hirschfield et al., 2021 [30]	Phase 3 RCT	Moderate	Larger sample size, but potential issues with reporting.

**Table 4:** Sensitivity analysis of ALP changes based on different NCDs.

NCD	Effect Size			Heterogeneity	
	SMD	95% CI	*p*-Value	I^2^	*p*-Value
Elafibranor	−2.02	(−3.11, −0.92)	0.0003	83%	0.003
Seladelpar	−4.42	(−6.24, −2.46)	<0.00001	96%	<0.00001
Fenofibrate	−1.04	(−1.65, −0.44)	0.0007	N/A	N/A
Bezafibrate	−3.37	(−4.00, −2.74)	<0.00001	N/A	N/A
Budesonide	−0.50	(−1.03, 0.03)	0.06	N/A	N/A
Saroglitazar	−5.01	(−6.26, 3.26)	<0.00001	0%	0.87

N/A, not applicable since the outcome was reported in only one study.

## Data Availability

Not applicable.
